# Editorial: Lignans: Insights Into Their Biosynthesis, Metabolic Engineering, Analytical Methods and Health Benefits

**DOI:** 10.3389/fpls.2020.630327

**Published:** 2021-01-12

**Authors:** Christophe F. Hano, Albena T. Dinkova-Kostova, Laurence B. Davin, John R. Cort, Norman G. Lewis

**Affiliations:** ^1^Laboratoire de Biologie des Ligneux et des Grandes Cultures, INRAE USC1328, Université d'Orléans, Chartres, France; ^2^COSM'ACTIFS, CNRS GDR3711, Chartres, France; ^3^Division of Cellular Medicine, Jacqui Wood Cancer Centre, Ninewells Hospital and Medical School, University of Dundee, Dundee, United Kingdom; ^4^Department of Pharmacology and Molecular Sciences, Johns Hopkins University School of Medicine, Baltimore, MD, United States; ^5^Department of Medicine, Johns Hopkins University School of Medicine, Baltimore, MD, United States; ^6^Institute of Biological Chemistry, Washington State University, Pullman, WA, United States; ^7^Earth and Biological Sciences Directorate, Pacific Northwest National Laboratory, Richland, WA, United States

**Keywords:** lignans, metabolic engineering, analytical method, biological activity, lignans metabolism

Lignans constitute a multifaceted group of phytochemicals widely distributed in terrestrial plant lineages (Ayres and Loike, 1990; Vassão et al., 2010). Lignans have important roles in plant physiology, development, and ecology (i.e., interactions and adaptations to ever-changing environments) (Burlat et al., 2001; Markulin et al., 2019). As their specialized metabolite nature might suggest, lignans have been implicated in plant defense protection against a variety of herbivores and microorganisms (Gang et al., 1999; Vassão et al., 2010; Seneviratne et al., 2015).

Reputable studies in the fields of human diet and/or nutritional care were initiated with the discovery of mammalian lignan (ML) formation from lignan-rich diets over the last decades (Axelson et al., 1982). Indeed, some lignans, aka “phytoestrogen” lignans, are converted into the MLs, enterodiol and enterolactone, by human gut microbiota upon their ingestion. These MLs have extensively described and discussed chemopreventive properties against various tumors (such as breast, colon, and prostate cancers) and/or cardiovascular disorders (Rietjens et al., 2017).

Critical analysis of the lignan research literature by Yeung et al. also revealed important features about trends in lignan research. Significantly, around 80% of lignan-related papers were published since 2000, of which about half of these were in 2010 or later; this clearly demonstrates a significant growth in interest over the last 20 years for this natural product family. Furthermore, the overall importance of flax (*Linum usitatissimum*), Schisandra (*Schisandra chinensis*), and Forsythia (*Forsythia x. intermedia*) is clearly evident in the literature analysis of lignans. Far from being limited solely to plant biology (around 20% of publications), many papers were centered on pharmacology (around one fourth) and chemistry (around one fourth). It should be noted that, in line with this observation, the current Research Topic herein includes studies on plant biology [i.e., biosynthesis of lignans and neolignans in flax (Bose et al.), chemistry] [e.g., extraction of sesame oil lignans (Michailidis et al.)], and pharmacology [e.g., pharmacological value of nordihydroguaiaretic acid (NDGA) and its (semi-)synthetic derivatives (Manda et al.)]. Yeung et al. also indicated a significant increase, in the most recently published publications, for studies based on the pharmacological importance of ingestion of lignans (e.g., secoisolariciresinol, lariciresinol, matairesinol, pinoresinol, medioresinol, and syringaresinol) in the diet, with particular emphasis on their cancer, cardiovascular disease, and diabetes prevention or antioxidant properties. However, the authors of this critical analysis also pointed out the need for more clinical trials to support beneficial effects and to establish optimal doses of lignan intake in humans, as such trials are estimated to contribute to only 0.2–1.1% of the literature reviewed.

Many lignans are formed by the oxidative coupling of E-coniferyl alcohol moieties. Lignans can share the same precursors as for lignins, the complex biopolymers that provide rigidity and support to vascular plants (Davin and Lewis, 2003). Understanding regulation of lignan biosynthesis is of particular importance for many applications. For instance, the use of plant tissue culture in cosmetics has gained renewed impetus in the last few decades, and the cosmetics industry is expected to expand to a turnover of several hundred billion US dollars per year. Interestingly, Bose et al. focused on *in vitro* culture of flax (*Linum usitatissimum*), a well-known rich source of lignans and neolignans, for its potent cosmetic properties. In-depth phytochemical study using UPLC-HRMS confirmed the high lignan and neolignan accumulation potential of this species, including 7 neolignans newly described, and their potential use in cosmetic applications. In particular, the study confirmed the importance of optimizing conditions of *in vitro* culture for a specific application.

Designing effective analytical methods for lignans also helps to gain new insights into natural lignan chemodiversity, evolution across the plant kingdom, as well as into the mechanism(s) of certain biological activities that remain elusive, and which require purified compounds for further study (Teponno et al., 2016). Moreover, extraction and purification steps are well-known limitations for the potential industrial use of certain lignans. Accordingly, due to their broad biological importance, a range of studies reported herein address isolation and purification of the lignans sesamin and sesamolin from sesame (*Sesamum indicum*), including their sourcing from sesame seed and sesame seed-derived products. Just a few previous works have reported producing both of these two lignans in high quantity and purity using a low cost, fast, methodology. That is, Michailidis et al. describe an integrated method for the recovery of sesame and sesamolin from sesame oil, with a purity of more than 95%, using centrifugal partition extraction. These purified compounds were then further tested for their tyrosinase, elastase, collagenase, and inhibition function of hyaluronidase in order to assess their cosmetic properties.

The path to the market of a natural product can often be lengthy and sometimes involves the removal of significant disadvantages, such as low solubility or adverse toxicity (Cragg and Newman, 2001). The most commonly known lignan example is podophyllotoxin, which is medicinally used worldwide as a starting compound for semi-synthesis of potent anticancer drugs that inhibit topoisomerase II (Cragg and Newman, 2001). Moreover, the review paper herein by Manda et al. sheds light on nordihydroguaiaretic acid (NDGA), another lead lignan medicinal compound, and a clear example of all these considerations. NDGA is a phenolic lignan from the creosote bush (*Larrea tridentata*), found in deserts of Mexico and the United States. It has long been used in traditional medicine for treating various diseases, including cancer, renal, cardiovascular, immunological, and neurological disorders, and even aging. The review encompasses current knowledge of NDGA uses, including its targets and side-effects and its synthetic analogs as potential therapeutic agents. In particular, preclinical studies in cell culture and rodents suggest that NDGA is a promising drug for the prevention or treatment of many chronic diseases and cancers, largely due to its direct (scavenging of reactive oxygen species) and indirect (activation of endogenous antioxidant responses mediated by transcription factor Nrf2) antioxidant effects. However, high concentrations of NDGA can also be cytotoxic. In recent years, production of NDGA analogs, some being more potent and target-selective, and exhibiting lower toxicity due to the prevention of the conversion of the catechol functionality to a quinone, has given new impetus to this area. These efforts have culminated in the development of tetra-*O*-methyl nordihydroguaiaretic acid (Terameprocol), currently used in several cancer clinical trials. Interestingly, some NDGA analogs are also promising in treatment of neurodegenerative disorders and metabolic syndrome.

In summary, *in planta* lignan biosynthesis and functions, together with their improved extraction procedures and/or health benefits, provide exciting new frontiers for scientists from numerous fields of expertise for further research. It is anticipated that the papers herein on this Research Topic have the benefit of shedding new light on this family of natural products, the interest in which has been increasing over the last 20 years, whether in plant science, chemistry or pharmacology.

## Author Contributions

All authors wrote, revised, and approved the final version of the manuscript.

## Conflict of Interest

The authors declare that the research was conducted in the absence of any commercial or financial relationships that could be construed as a potential conflict of interest.
